# A unique set of complex chromosomal abnormalities in an infant with myeloid leukemia associated with Down syndrome

**DOI:** 10.1186/s13039-017-0335-3

**Published:** 2017-09-11

**Authors:** Daiane Correa de Souza, Amanda Faria de Figueiredo, Daniela R. Ney Garcia, Elaine Sobral da Costa, Moneeb A.K. Othman, Thomas Liehr, Eliana Abdelhay, Maria Luiza Macedo Silva, Teresa de Souza Fernandez

**Affiliations:** 1 Cytogenetic Laboratory, Bone Marrow Transplantation Center, National Cancer Institute (INCA), Praça Cruz Vermelha no. 23, 6° andar. Centro, CEP, Rio de Janeiro, RJ 20230-130 Brazil; 2 Pediatric and Puericulture Martagão Gesteira Institute, Federal University of Rio de Janeiro, Rio de Janeiro, 21941-590 Brazil; 3 Jena University Hospital, Friedrich Schiller University, Institute of Human Genetics, Kollegiengasse 10, 07743 Jena, Germany

**Keywords:** Myeloid leukemia, Down syndrome, Unreported chromosomal abnormalities, Complex karyotype, Molecular cytogenetic, Prognosis

## Abstract

**Background:**

Children with Down syndrome (DS) have an enhanced risk of developing acute leukemia, with the most common subtype being acute megakaryoblastic leukemia (AMKL). Myeloid leukemia in Down syndrome (ML-DS) is considered a disease with distinct clinical and biological features. There are few studies focusing on the clonal cytogenetic changes during evolution of ML-DS.

**Case presentation:**

Here, we describe a complex karyotype involving a previously unreported set of chromosomal abnormalities acquired during progression of ML-DS in an infant boy: derivative der(1)t(1;15)(q24;q23), translocation t(4;5)(q26;q33) and derivative der(15)t(7;15)(p21;q23). Different molecular cytogenetic probes and probesets including whole chromosome painting (WCP) and locus specific probes, as well as, multicolor-FISH and multicolor chromosome banding (MCB) were performed in order to characterize the chromosomal abnormalities involved in this complex karyotype. The patient was treated according to the acute myeloid leukemia-Berlin-Frankfurt-Munich-2004 (AML-BFM 2004) treatment protocol for patients with Down syndrome; however, he experienced a poor clinical outcome.

**Conclusion:**

The molecular cytogenetic studies performed, allowed the characterization of novel chromosomal abnormalities in ML-DS and possible candidate genes involved in the leukemogenic process. Our findings suggest that the complex karyotype described here was associated with the poor prognosis.

## Background

Children with Down syndrome (DS) have a higher risk of developing acute leukemia than children without DS, and the most common subtype is acute megakaryoblastic leukemia (AMKL) [1]. Myeloid leukemia in Down syndrome (ML-DS) is a disease with distinct clinical and biological features, encompassing both myelodysplastic syndrome (MDS) and acute myeloid leukemia (AML) [2, 3]. ML-DS can be preceded by transient myeloproliferative disease (TMD) in newborns. Although TMD spontaneously disappears in most cases, approximately 20% of children diagnosed with TMD develop ML-DS [4]. ML-DS is characterized by a higher occurrence in young age, low number of leukemic blasts in the bone marrow, somatic mutations in *GATA-1* (a transcription factor that regulates the differentiation of megakaryocyte and erythrocyte precursors) and a better clinical outcome when treated with reduced-intensity chemotherapy protocols [5].

Most cytogenetic studies on DS-related leukemia have been single case reports or relatively small series. However, several studies have shown that the karyotypic patterns of ML-DS are different from those observed in AML of children without DS, e.g. translocations t(8;21), t(15;17), t(9;11), inversion inv.(16), as well as AMKL associated translocations t(1;22) and t(1;3) [1, 5, 6]. The most frequent chromosomal alterations associated with ML-DS are: duplication dup(1q), deletion del(6q), del(7p), dup(7q), trisomy +8, +11, del(16q) and +21. According to Forestier and colleagues [1], the types and frequencies of chromosomal changes occurring in addition to the constitutional +21 in ML-DS may provide important clues to the pathogenesis of acute leukemia in such patients.

Even though, the importance of cytogenetic alterations in classification and risk stratification of non-DS-AML is well recognized, e.g. in World Health Organization classification, there is limited information on cytogenetic alterations and their prognostic impact in ML-DS [1–5]. In a previous collaborative international AML-BFM Group Study, Blink and colleagues [5] described the first study on the prognostic impact of cytogenetic groups in 358 patients with ML-DS. In this study, the cytogenetic risk groups were: normal karyotype, trisomy 8, loss of chromosomes 5 and 7, trisomy 21, dup(1q), del(16q) and other chromosomal alterations. However, complex karyotypes were not categorized. There are a few studies focusing on the clonal cytogenetic changes during the evolution of ML-DS and the prognostic impact of a complex karyotype in ML-DS [4, 7–10]. The role of acquired chromosomal abnormalities (ACAs) in the progression of ML-DS has been discussed [10]. Some studies indicated that ACAs appear to be a risk factor for progression of disease [9, 10]. However, other studies did not observe this association [4, 5].

Here, we describe a complex karyotype with yet unreported chromosomal abnormalities in a 1-year-old boy presenting ML-DS. These chromosomal abnormalities were defined by molecular cytogenetic approaches and this complex karyotype was associated with a poor prognosis. Taken together with data from the literature, this may be supportive to include complex karyotypes in ML-DS as an additional adverse risk factor in classification and risk stratification of ML-DS.

## Case presentation

A DS infant boy (one year of age) with a history of thrombocytopenia was referred to Martagão Gesteira Institute for clinical investigation, Rio de Janeiro, RJ, Brazil. Peripheral blood values were: hemoglobin 8.3 g/dl (age-adjusted range: 13.5–18.0 g/dl), platelet count 10 × 10^9^/l (150–400 × 10^9^/l) and white blood cell count of 48 × 10^9^/l (age-adjusted range: 4–10 × 10^9^/l). Morphologic evaluation of the bone marrow revealed hypocellularity (with granulocytic population decreased), the presence of dysplasia in the erythroid and megakaryocytic lineages, suggesting a diagnosis of ML-DS (myelodysplastic syndrome). Flow cytometry analysis of bone marrow (BM) cells showed a maturation block in granulocytic and monocytic lineages with high expression of CD7 and CD56 in the monocytic maturation. Cytogenetic analysis of bone marrow cells using G-banding showed: 47,XY,add(7)(p?),add(15)(q?), +21c [14]/47,XY,+21c [14].

**Fig. 1:**
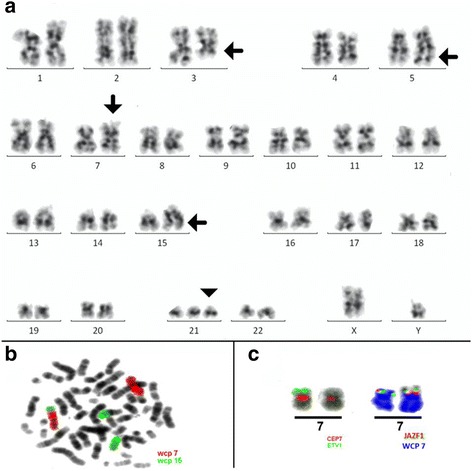
**a** G-banding showing the complex karyotype, chromosomal aberrations are pointed by the arrows; (**b**) Whole chromosome paints (WCP) for chromosomes 7 and 15 confirmed the unbalanced nature of the translocations observed in G-banding; (**c**) Application of ETV1/CEP7 and JAZF1 break-apart combined with WCP for chromosome 7 probes narrowed the breakpoint down to 7p21 and showed the ETV1 gene deletion

**Fig. 2:**
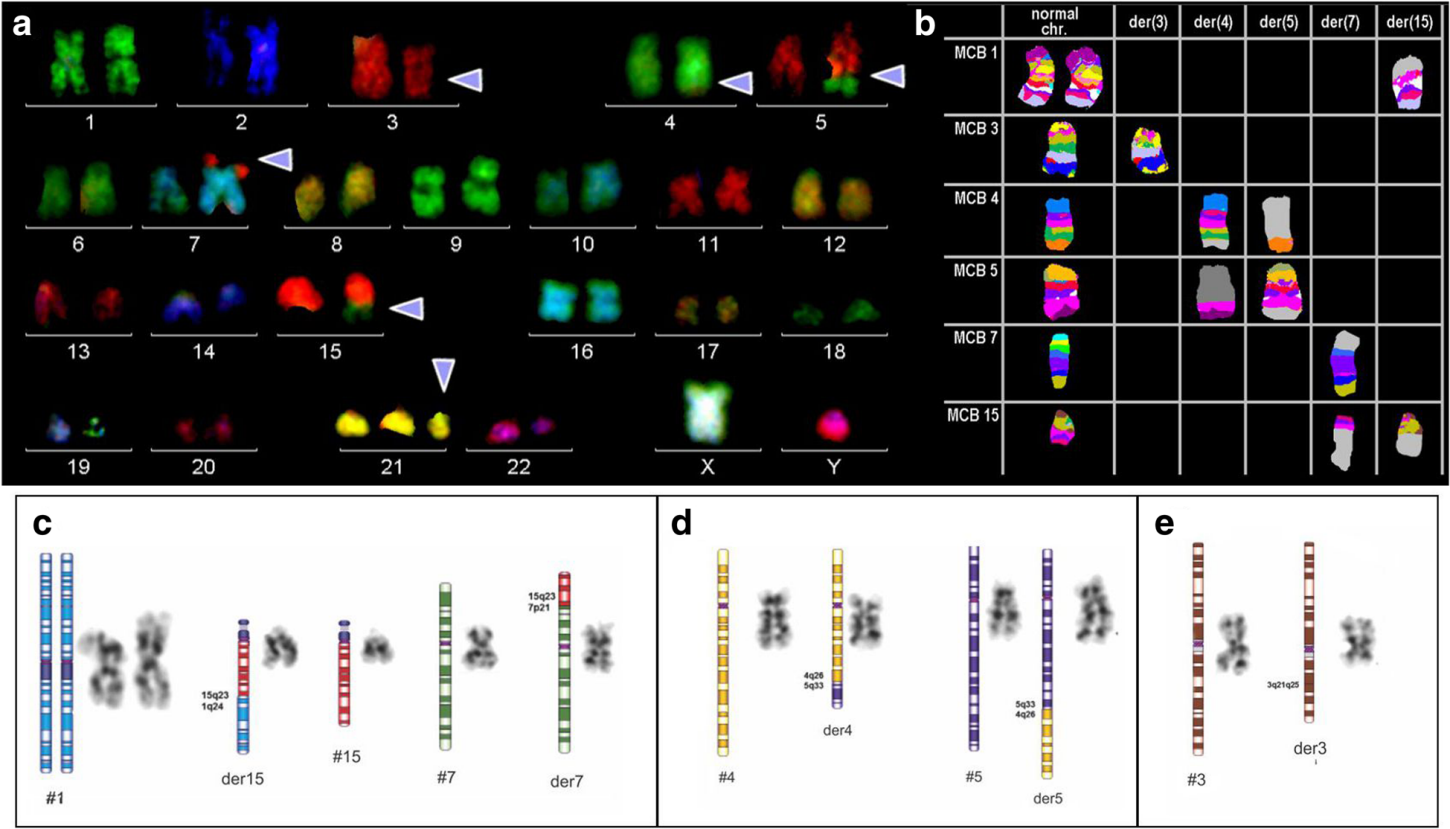
**a** M-FISH results showing the trisomy 21 and the involvement of chromosomes 3, 4, 5, 7 and 15 in this complex karyotype; (**b**) Pseudocolor depiction of MCB for the chromosomes involved in the complex rearrangement; (**c**-**e**) Schematic drawings of the rearranged chromosomes compared to their formats in G-banding

Three months later, the patient presented with 51.8% of blast cells and the following immunophenotype: CD45^lo/+^, CD117^hi^, HLADR^−/+(40%)^, CD13^−/+(40%)^, CD33^+^, CD36^++^, CD34^−/+(1%)^, IREM2^−/+(10%)^, CD71^+lo^, CD11b^−^, CD16^−^, CD64^−^, CD14^−^, CD15^−^, MPO^−^, CD123^−^, CD9^−^, CD41a^−^, TdT^−^, CD7^−^, CD56^−^, cyCD3^−^, CD3^−^, CD19^−^ and cyCD79a^−^, compatible with ML-DS (AML secondary from MDS). Classical cytogenetic analysis was performed, during AML secondary from MDS, using bone marrow cells and G-banding. This analysis revealed a clonal karyotypic evolution with the complex karyotype:

47,XY,t(3;5)(q21;q32),add(7)(p?),add(15)(q?),+21c [14]/ 47,XY,add(7)(p?),add(15)(q?),+21c [5]/47,XY,+21c [3] (Fig. 1a). Different molecular cytogenetic approaches were performed in order to characterize the chromosomal alterations. Initially, we used the whole chromosome painting (WCP) probes for chromosomes 7 and 15 (Fig. 1b). The karyotype was defined as: 47,XY,del(3)(q21),add(5q),der(7)t(7;15)(p15;q21q26),add(15)(q21),+21c [18]/47,XY, der(7)t(7;15)(p15;q21q26),add(15)(q21),+21c [5]/47,XY,+21c [2]. Afterward, more specific probes were used to characterize genes possibly involved in the chromosomal rearrangements: SPEC *JAZF* in 7p15*,* SPEC *ETV1* in 7p21, SPEC *SOX2* in 3q26, and SPEC *VHL* in 3p25 (Zytovison, Bremerhaven, Germany) and subtelomeric probe for 7pter (Abbott/Vysis, Göttingen, Germany). Thus, we could narrow down the breakpoint between deletion 7p21 and 7p15, revealing the deletion of the *ETV1* gene (Fig. 1c). With multicolor-FISH, the complex karyotype was confirmed and refined as 47,XY,der(3)del(3)(p),der(4)t(4;5),der(7)t(7;15),+21c (Fig. 2a). Multicolor banding (MCB) was performed for chromosomes 1, 3, 4, 5, 7 and 15 (Fig. 2b), as described by Liehr and colleagues [11]. The chromosomal rearrangements and the breakpoints involved in derivative chromosomes 1, 3, 4, 5, 7 and 15 were defined (Fig. 2c-e). The final karyotype was characterized as: 46,XY,der(1)t(1;15)(q24;q23),del(3)(q21q25),t(4;5)(q26;q33), del(7)(p21),der(15)t(7;15)(p21;q23),+21c. The karyotypes were described according to the International System for Human Cytogenetic Nomenclature [12].

The patient was treated with AML-BFM 2004 protocol, arm designed for DS. At the end of induction therapy, he had 5% of blasts in bone marrow. After the first two blocks of high doses intensification, he presented with 20% of blasts in peripheral blood, sepsis and died.

## Discussion and conclusions

DS leukemogenesis is suggested to be a multistep process in which progenitor cells acquire multiple genetic lesions during progression to acute leukemia. The first event is trisomy 21, but it is not sufficient for malignant cell expansion [13, 14]. At cytogenetic level, the present case illustrates this concept, since during leukemic evolution the patient acquired new cytogenetic abnormalities resulting in a complex karyotype. Present since the initial diagnosis ML-DS (MDS phase), the translocation t(7;15)(p21;q23) was associated with a deletion of the *ETV1* gene in 7p21 and a rearrangement of *MAP2K5* (mitogen-activated protein kinase 5) located in 15q23. The *ETV1* (ETS-translocation variant 1) gene belongs to the ETS (erythroblastosis virus E26 transforming sequences) family of transcription factors. The ETS proteins regulate many target genes that modulate biological processes like cell growth, angiogenesis, migration, proliferation and differentiation [15]. Pathologically, the ETV1 protein is aberrantly expressed through chromosomal translocations in a subset of solid tumors, e.g. prostate, melanoma, gastrointestinal stromal tumors [16]. Interestingly, in this study, the *ETV1* gene was deleted, so probably altering the signaling pathways of biological processes which are thought to contribute to the leukemogenesis. Furthermore, MAPK pathways play critical roles in a wide variety of cancer types, from hematologic malignancies to solid tumors [17]. To the best of our knowledge, this translocation has not been described yet in ML-DS.

Besides translocation t(7;15), a derivative der(1)t(1;15)(q24;q23) was also present at the initial stage of ML-DS. Partial trisomy of the long arm of chromosome 1, by means of duplication, has been recurrently reported and appears to represent a non-random chromosomal abnormality in ML-DS [1, 5, 18]. So, we hypothesized that the co-existence of translocation t(7;15)(p21;q23) and derivative der(1)t(1;15)(q24;q23) could be drivers of genetic aberrations, that block normal myeloid differentiation, potentially inducing the leukemic evolution. During this leukemogenesis process, the progenitor cells acquired other chromosomal abnormalities.

When leukemic infiltration was diagnosed, two additional chromosomal abnormalities were observed during disease progression: deletion del(3)(q21q25) and translocation t(4;5)(q26;q33). Translocations or inversions involving 3q21 and 3q26 are associated with high-risk in AML and these patients usually present a poor prognosis [19]. The translocation t(4;5)(q26;q33) has not yet been described in an ML-DS before. Interestingly, the N-deacetylase/N-sulfotransferase (*NDST4*) gene, is located in 4q26. The encoded enzyme has a dual function, i.e. processing glucosamine and heparan polymers, the latter being key components of cell microenvironment, playing an important role in cell-cell interactions and adhesion. Bone marrow microenvironment has been implicated as a source of chemoresistance and disease relapse [20]. In the second breakpoint 5q33-q34, there are individual genes such as *EGR1*, *CSF1R*, and *RPS14* that may contribute to malignant transformation [21]. This new complex karyotype originated during the evolution of the disease, associated with poor clinical outcome presented by our patient, reinforces the importance of routinely karyotyping of ML-DS.

Our data have confirmed and extended prior knowledge that ML-DS is cytogenetically characterized mainly by a relatively high frequency of copy number alterations [18]. In the present work, we described a complex karyotype characterized by chromosomal translocations (balanced and unbalanced), suggesting other mechanisms involved in the appearance of these chromosomal abnormalities. Trisomies are quite characteristic for ML-DS, as trisomy 8 and 11, suggesting that DS patients may be more susceptible to non-disjunctional events during cell division [1, 22].

The present case exemplifies the clonal karyotypic evolution being typical for ML-DS and demonstrated the impact of ACAs during the evolution of ML-DS. Thus, defining the cytogenetic and molecular characteristics of disease progression in ML-DS is important to understand the pathogenesis of ML-DS. For this purpose, we emphasize the utility of multicolor FISH, once the involvement of chromosome 1 was only detected using this molecular cytogenetic method. Therefore, further molecular studies involving a larger number of patients are needed to clarify the dilemma of the cytogenetic impact in ML-DS prognosis, making possible the incorporation of the cytogenetic information of leukemia associated with DS in the risk group stratification.

In summary, our study suggests that the leukemogenic process was triggered by the accumulation of ACAs. The molecular cytogenetic methods applied in this highly complex karyotype allowed the characterization of chromosomal regions with high resolution and provided the ability to identify possible candidate genes involved in the leukemogenic process, giving new insights into the biology of ML-DS. As the correlation of cytogenetic changes with the development of the disease and its prognosis in ML-DS is not as clear yet as in children without DS, our study shows that ACAs were clearly associated with the evolution of the disease, resulting in a complex karyotype; thus, the latter may be supportive of including complex karyotypes in ML-DS as an adverse risk factor in classification and risk stratification of ML-DS in near future.

## Abbreviations

+: trisomy
ACAs: Acquired chromosomal abnormalities
AMKL: Acute megakaryoblastic leukemia
AML: Acute myeloid leukemia
del: deletion
der: derivative
DS: Down syndrome
dup: duplication
FISH: Fluorescence *in situ* hybridization
inv.: inversion
MDS: Myelodysplastic syndrome
ML: Myeloid leukemia
ML-DS: Myeloid leukemia of Down syndrome
SPEC: Spectrum
t: translocation
TMD: Transient myeloproliferative disease
WCP: Whole chromosome painting
